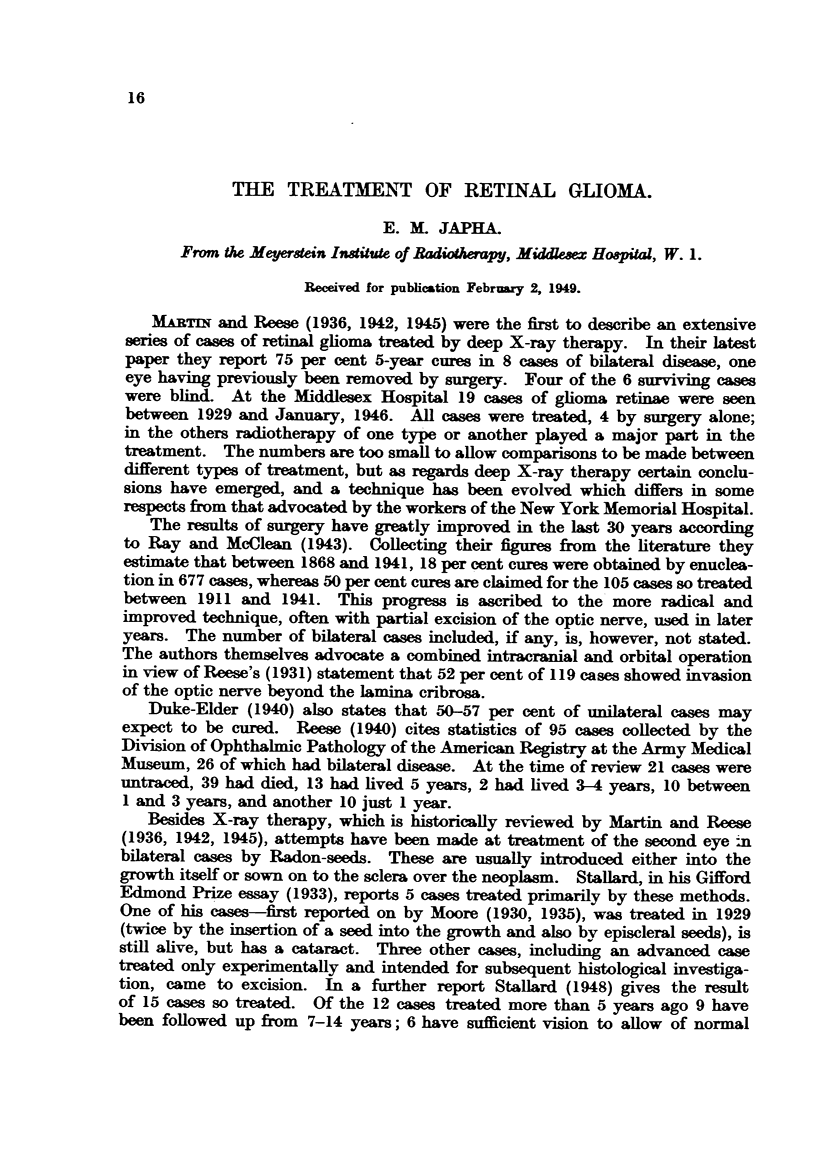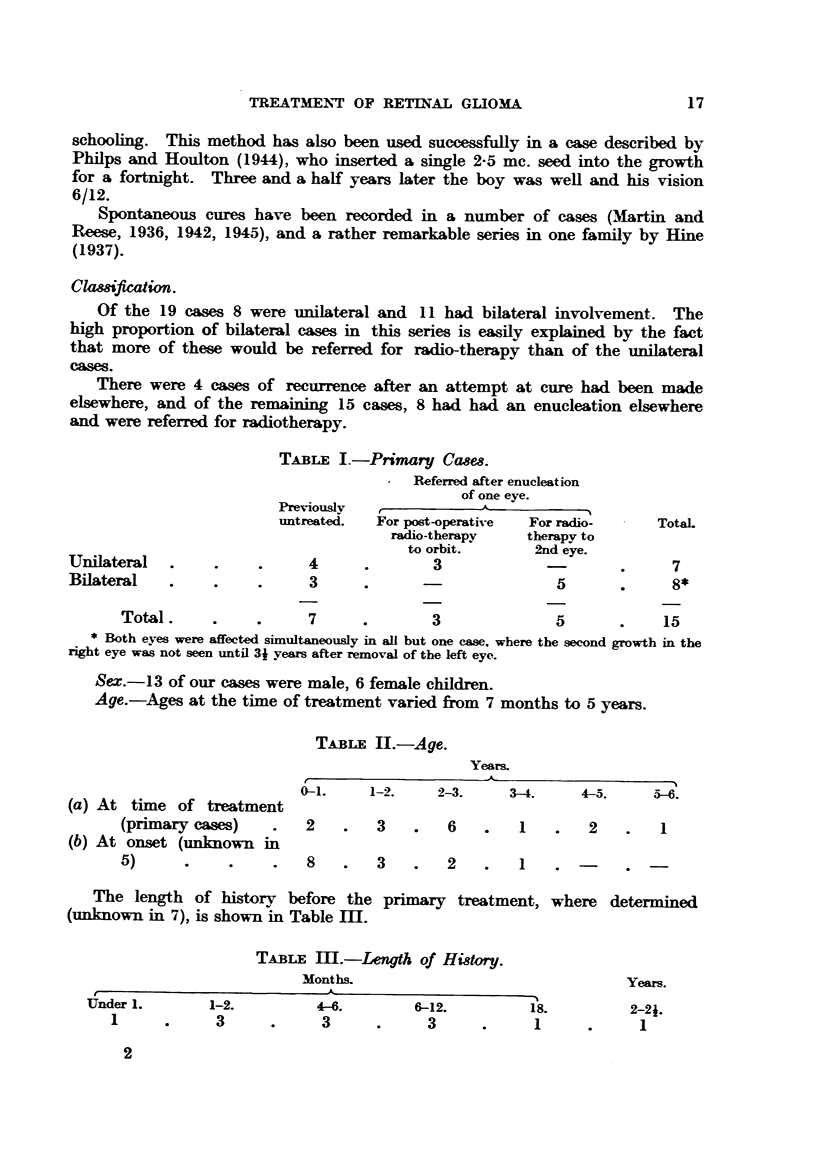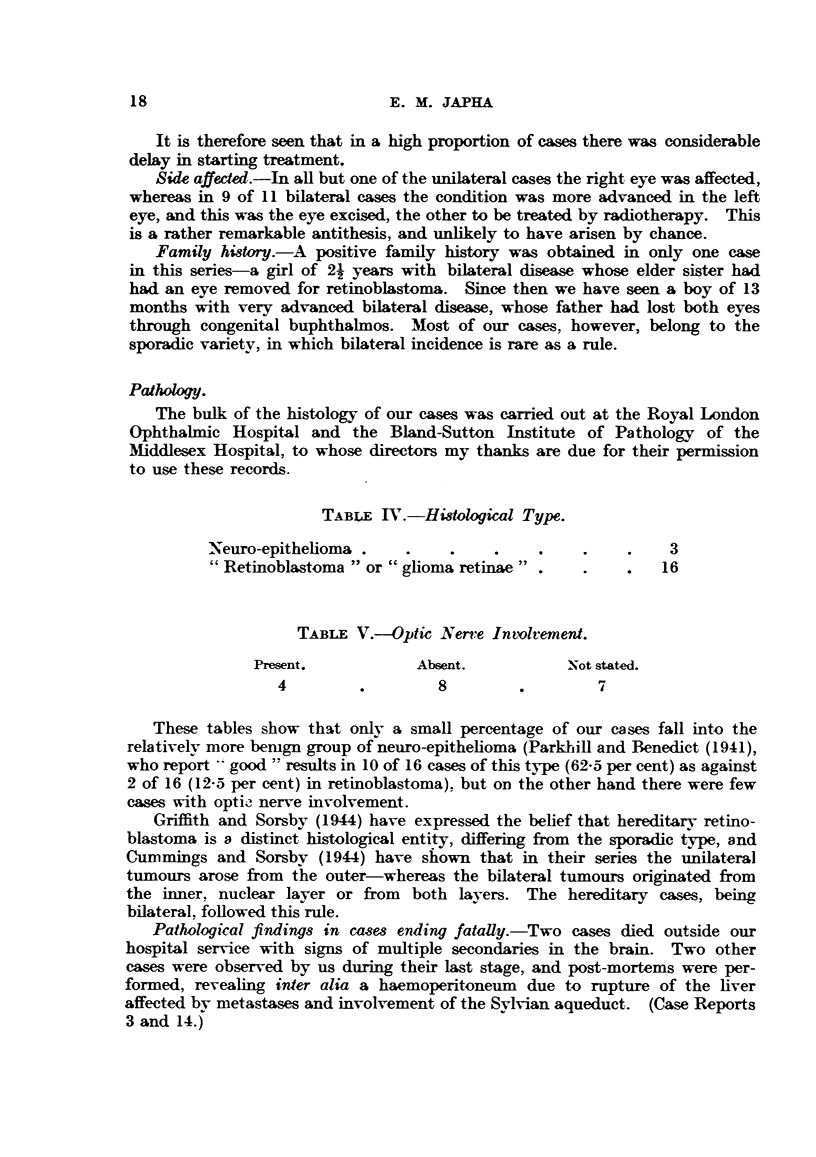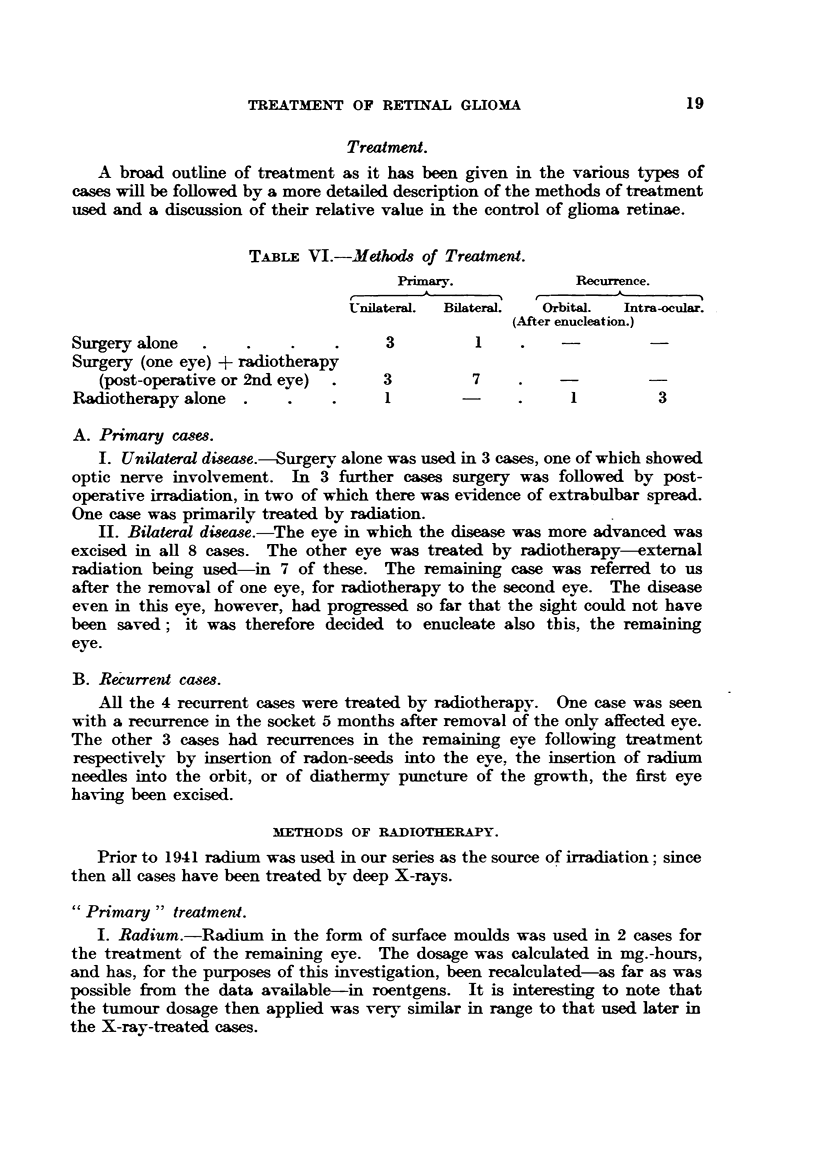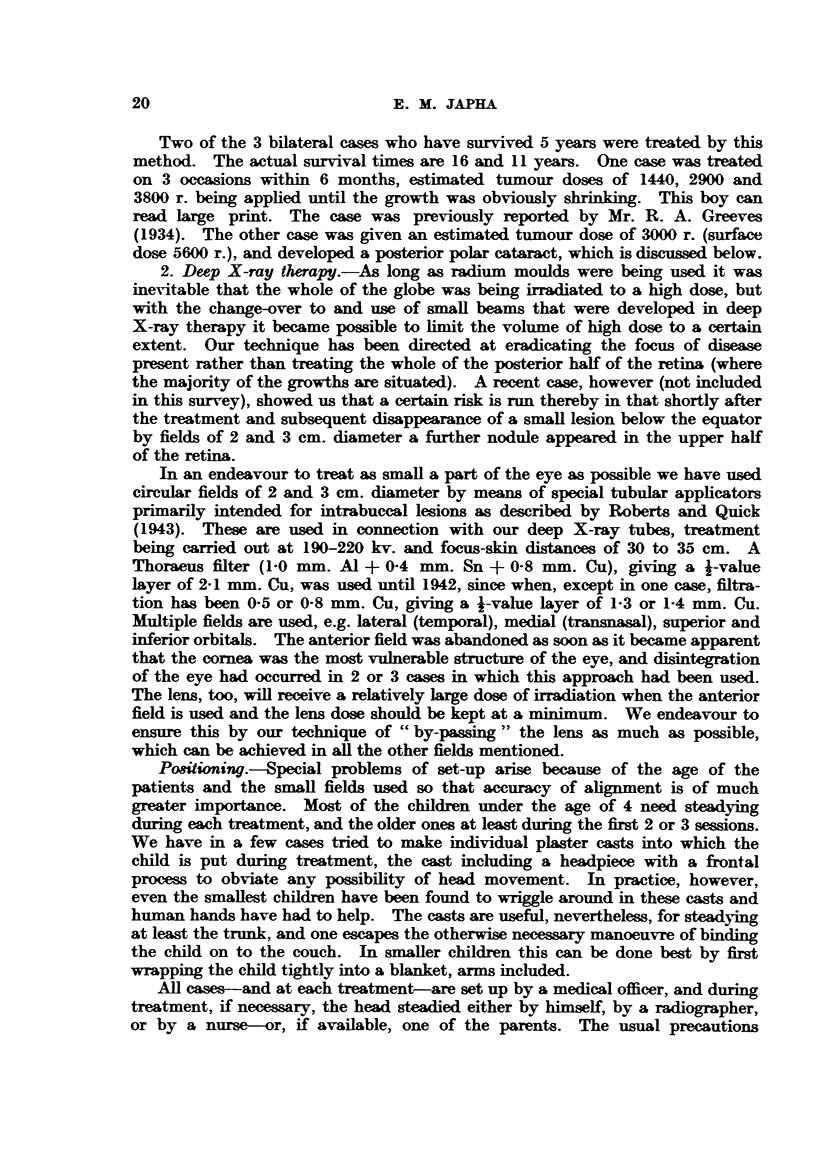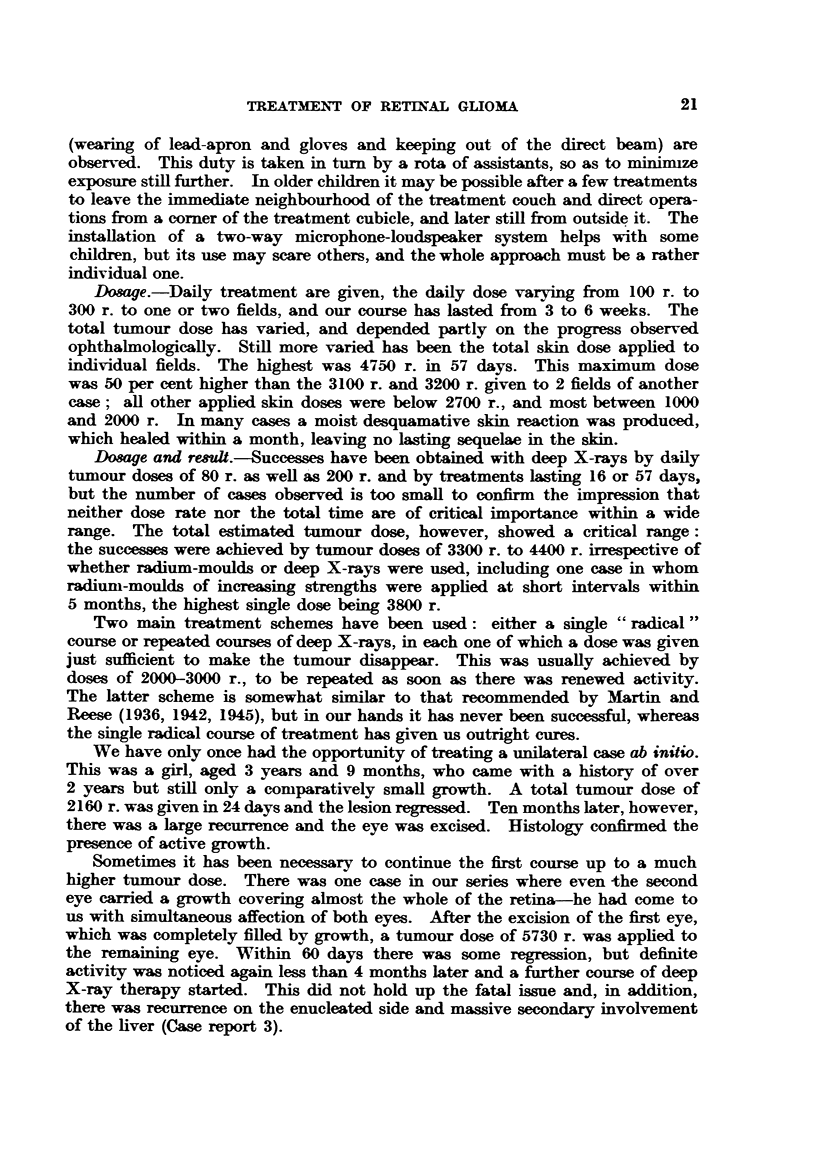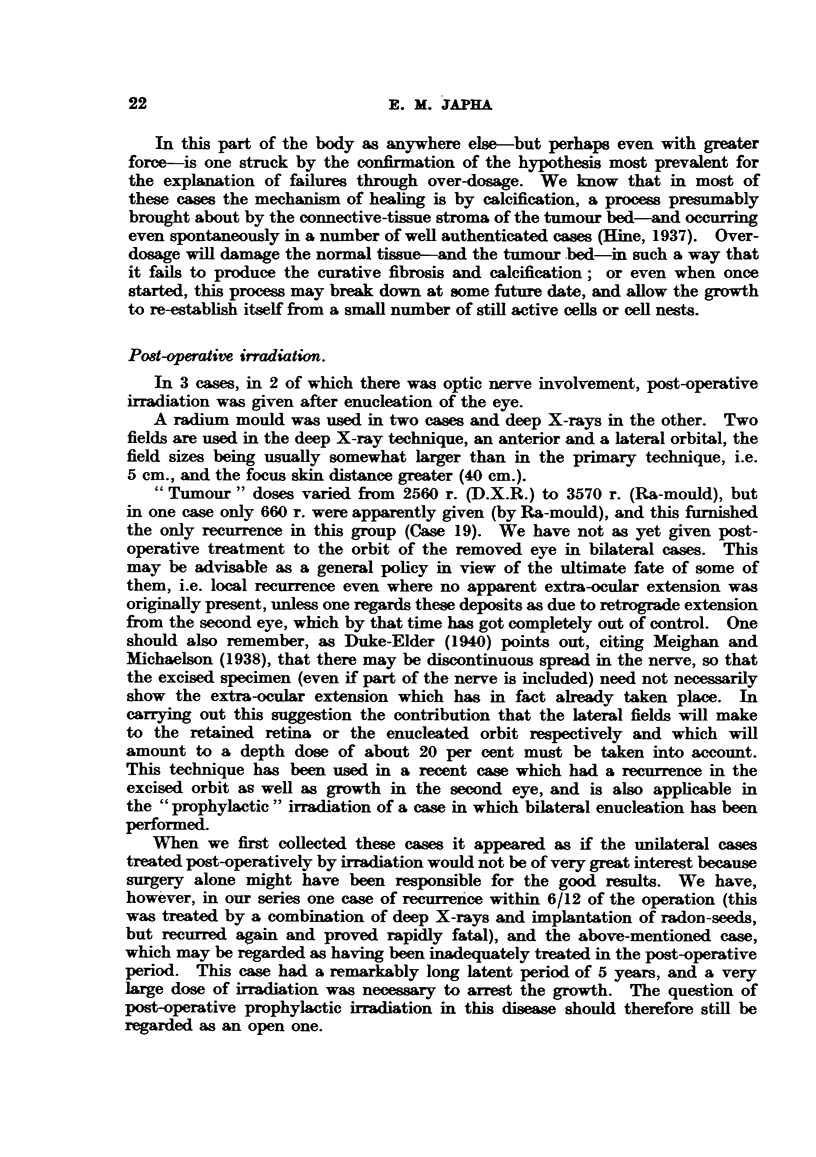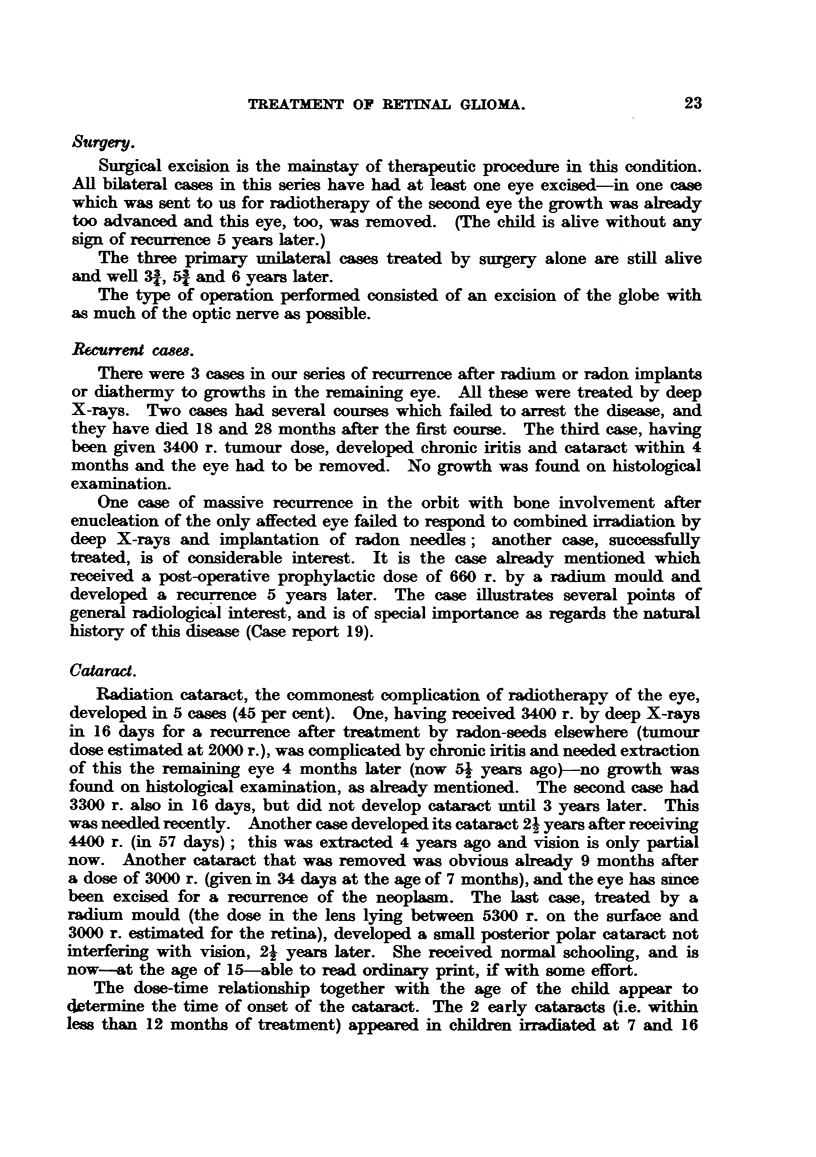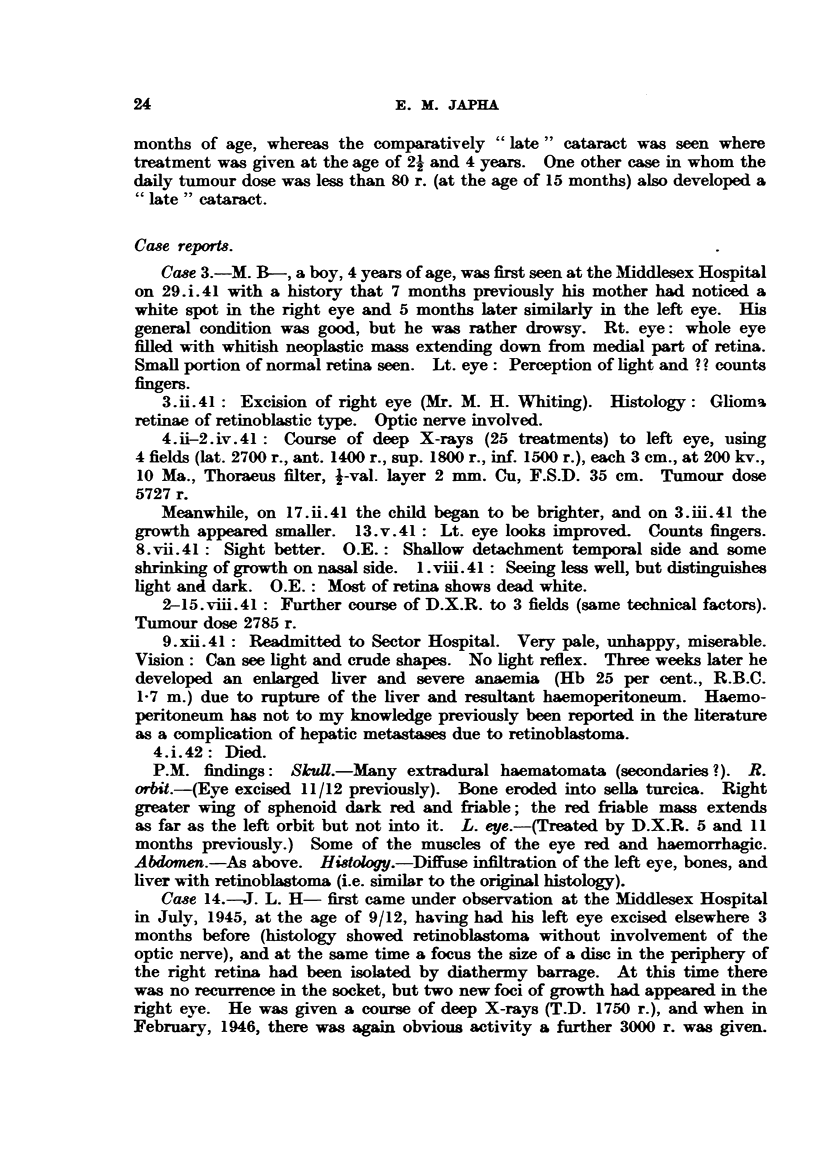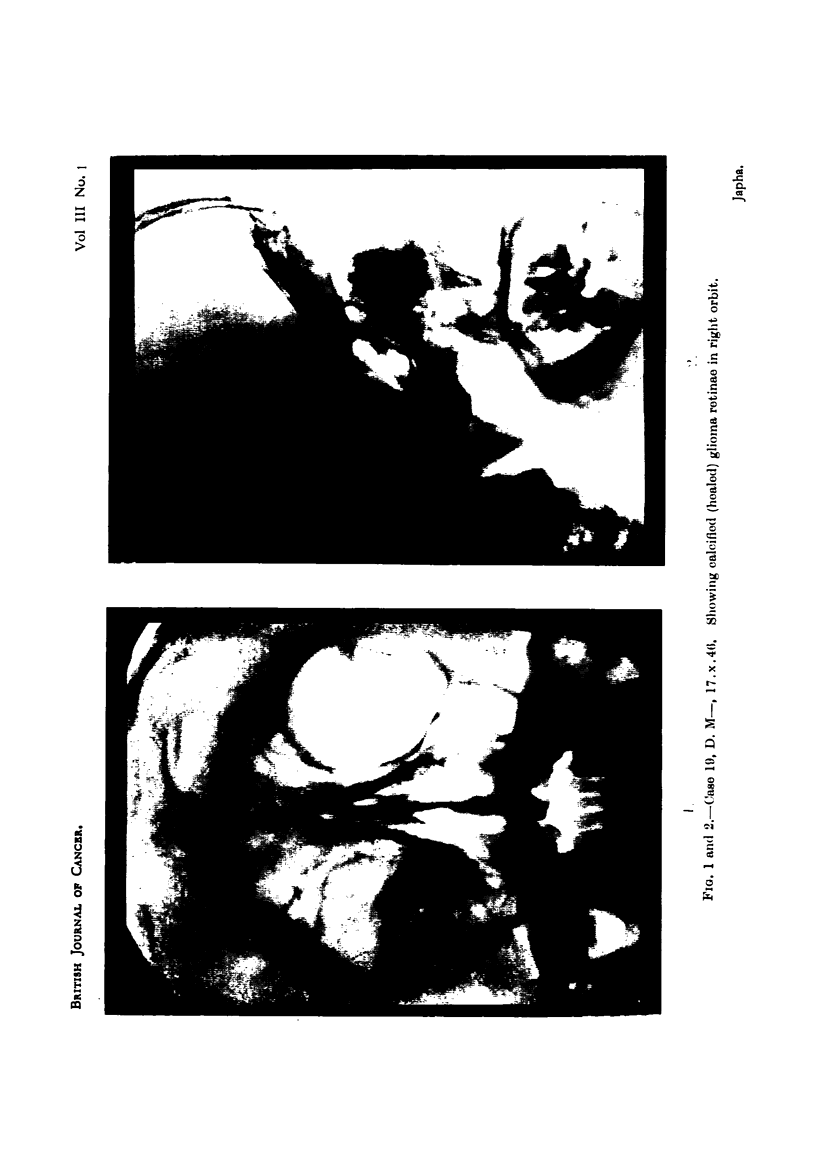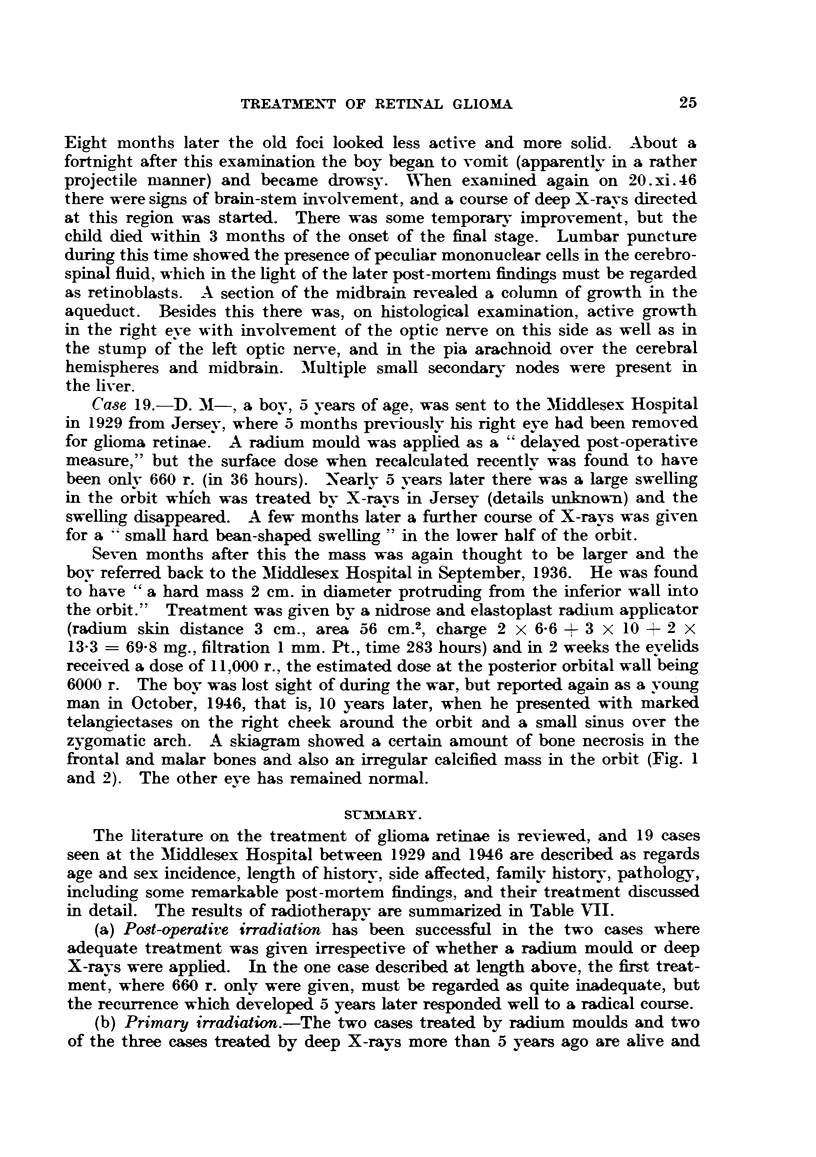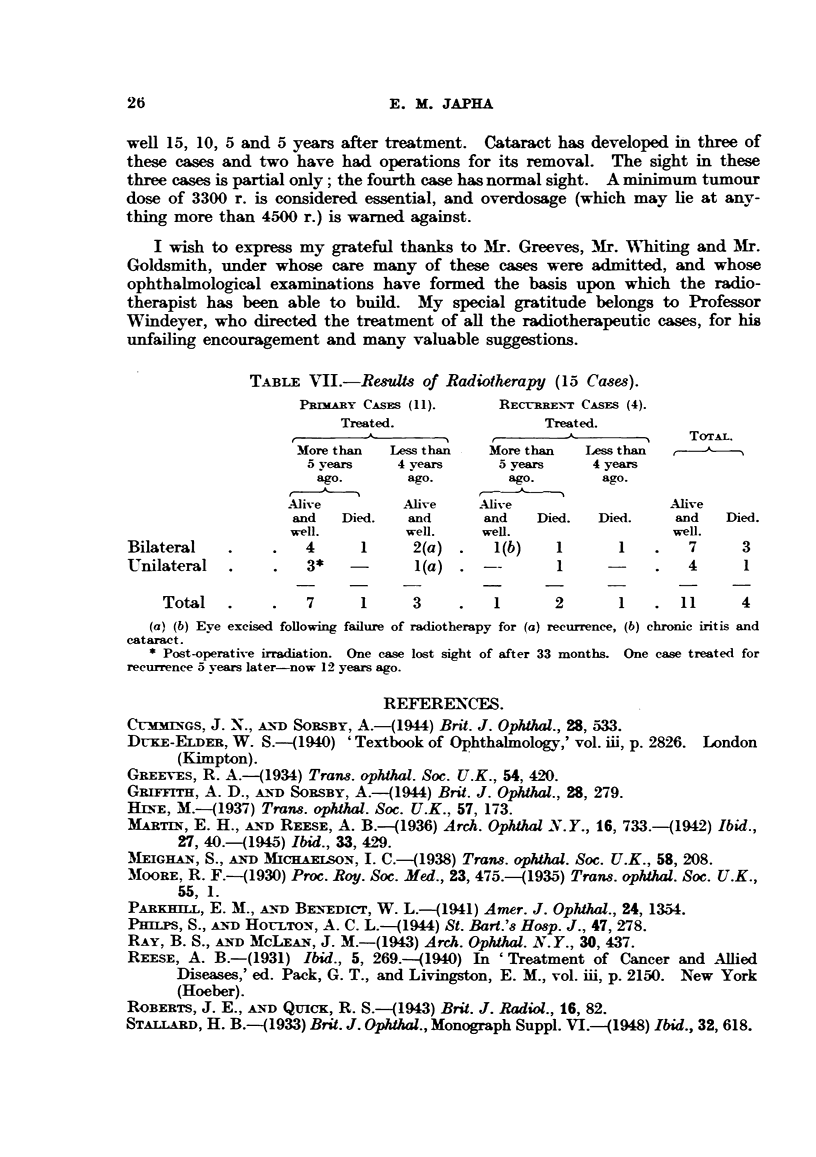# The Treatment of Retinal Glioma

**DOI:** 10.1038/bjc.1949.2

**Published:** 1949-03

**Authors:** E. M. Japha

## Abstract

**Images:**


### THE TREATMENT OF RETINAL GLIOMA.

E. M. JAPHA.

*From the Meyerstein Institute of Radiotherapy, Middlesex Hospital, W. 1.*

Received for publication February 2, 1949.

MARTIN and Reese (1936, 1942, 1945) were the first to describe an extensive series of cases of retinal glioma treated by deep X-ray therapy. In their latest paper they report 75 per cent 5-year cures in 8 cases of bilateral disease, one eye having previously been removed by surgery. Four of the 6 surviving cases were blind. At the Middlesex Hospital 19 cases of glioma retinae were seen between 1929 and January, 1946. All cases were treated, 4 by surgery alone; in the others radiotherapy of one type or another played a major part in the treatment. The numbers are too small to allow comparisons to be made between different types of treatment, but as regards deep X-ray therapy certain conclusions have emerged, and a technique has been evolved which differs in some respects from that advocated by the workers of the New York Memorial Hospital.

The results of surgery have greatly improved in the last 30 years according to Ray and McClean (1943). Collecting their figures from the literature they estimate that between 1868 and 1941, 18 per cent cures were obtained by enucleation in 677 cases, whereas 50 per cent cures are claimed for the 105 cases so treated between 1911 and 1941. This progress is ascribed to the more radical and improved technique, often with partial excision of the optic nerve, used in later years. The number of bilateral cases included, if any, is, however, not stated. The authors themselves advocate a combined intracranial and orbital operation in view of Reese's (1931) statement that 52 per cent of 119 cases showed invasion of the optic nerve beyond the lamina cribrosa.

Duke-Elder (1940) also states that 50–57 per cent of unilateral cases may expect to be cured. Reese (1940) cites statistics of 95 cases collected by the Division of Ophthalmic Pathology of the American Registry at the Army Medical Museum, 26 of which had bilateral disease. At the time of review 21 cases were untraced, 39 had died, 13 had lived 5 years, 2 had lived 3–4 years, 10 between 1 and 3 years, and another 10 just 1 year.

Besides X-ray therapy, which is historically reviewed by Martin and Reese (1936, 1942, 1945), attempts have been made at treatment of the second eye in bilateral cases by Radon-seeds. These are usually introduced either into the growth itself or sown on to the sclera over the neoplasm. Stallard, in his Gifford Edmond Prize essay (1933), reports 5 cases treated primarily by these methods. One of his cases—first reported on by Moore (1930, 1935), was treated in 1929 (twice by the insertion of a seed into the growth and also by episcleral seeds), is still alive, but has a cataract. Three other cases, including an advanced case treated only experimentally and intended for subsequent histological investigation, came to excision. In a further report Stallard (1948) gives the result of 15 cases so treated. Of the 12 cases treated more than 5 years ago 9 have been followed up from 7–14 years; 6 have sufficient vision to allow of normal schooling. This method has also been used successfully in a case described by Philps and Houlton (1944), who inserted a single 2·5 mc. seed into the growth for a fortnight. Three and a half years later the boy was well and his vision 6/12.

Spontaneous cures have been recorded in a number of cases (Martin and Reese, 1936, 1942, 1945), and a rather remarkable series in one family by Hine (1937).

*Classification.*

Of the 19 cases 8 were unilateral and 11 had bilateral involvement. The high proportion of bilateral cases in this series is easily explained by the fact that more of these would be referred for radio-therapy than of the unilateral cases.

There were 4 cases of recurrence after an attempt at cure had been made elsewhere, and of the remaining 15 cases, 8 had had an enucleation elsewhere and were referred for radiotherapy.

TABLE I.—*Primary Cases.*

| | Previously untreated. | Referred after enucleation of one eye. For post-operative radio-therapy to orbit. | For radio-therapy to 2nd eye. | Total. |
|---|---|---|---|---|
| Unilateral | 4 | 3 | — | 7 |
| Bilateral | 3 | — | 5 | 8* |
| Total | 7 | 3 | 5 | 15 |

* Both eyes were affected simultaneously in all but one case, where the second growth in the right eye was not seen until 3½ years after removal of the left eye.

*Sex.*—13 of our cases were male, 6 female children.

*Age.*—Ages at the time of treatment varied from 7 months to 5 years.

TABLE II.—*Age.*

| | Years. 0–1. | 1–2. | 2–3. | 3–4. | 4–5. | 5–6. |
|---|---|---|---|---|---|---|
| (*a*) At time of treatment (primary cases) | 2 | 3 | 6 | 1 | 2 | 1 |
| (*b*) At onset (unknown in 5) | 8 | 3 | 2 | 1 | — | — |

The length of history before the primary treatment, where determined (unknown in 7), is shown in Table III.

TABLE III.—*Length of History.*

| Months. Under 1. | 1–2. | 4–6. | 6–12. | 18. | Years. 2–2½. |
|---|---|---|---|---|---|
| 1 | 3 | 3 | 3 | 1 | 1 |

It is therefore seen that in a high proportion of cases there was considerable delay in starting treatment.

*Side affected.*—In all but one of the unilateral cases the right eye was affected, whereas in 9 of 11 bilateral cases the condition was more advanced in the left eye, and this was the eye excised, the other to be treated by radiotherapy. This is a rather remarkable antithesis, and unlikely to have arisen by chance.

*Family history.*—A positive family history was obtained in only one case in this series—a girl of 2½ years with bilateral disease whose elder sister had had an eye removed for retinoblastoma. Since then we have seen a boy of 13 months with very advanced bilateral disease, whose father had lost both eyes through congenital buphthalmos. Most of our cases, however, belong to the sporadic variety, in which bilateral incidence is rare as a rule.

*Pathology.*

The bulk of the histology of our cases was carried out at the Royal London Ophthalmic Hospital and the Bland-Sutton Institute of Pathology of the Middlesex Hospital, to whose directors my thanks are due for their permission to use these records.

TABLE IV.—*Histological Type.*

| | |
|---|---|
| Neuro-epithelioma . . . . . . . . | 3 |
| " Retinoblastoma " or " glioma retinae " . . . | 16 |

TABLE V.—*Optic Nerve Involvement.*

| Present. | Absent. | Not stated. |
|---|---|---|
| 4 . | 8 . | 7 |

These tables show that only a small percentage of our cases fall into the relatively more benign group of neuro-epithelioma (Parkhill and Benedict (1941), who report " good " results in 10 of 16 cases of this type (62·5 per cent) as against 2 of 16 (12·5 per cent) in retinoblastoma), but on the other hand there were few cases with optic nerve involvement.

Griffith and Sorsby (1944) have expressed the belief that hereditary retinoblastoma is a distinct histological entity, differing from the sporadic type, and Cummings and Sorsby (1944) have shown that in their series the unilateral tumours arose from the outer—whereas the bilateral tumours originated from the inner, nuclear layer or from both layers. The hereditary cases, being bilateral, followed this rule.

*Pathological findings in cases ending fatally.*—Two cases died outside our hospital service with signs of multiple secondaries in the brain. Two other cases were observed by us during their last stage, and post-mortems were performed, revealing *inter alia* a haemoperitoneum due to rupture of the liver affected by metastases and involvement of the Sylvian aqueduct. (Case Reports 3 and 14.)

### *Treatment.*

A broad outline of treatment as it has been given in the various types of cases will be followed by a more detailed description of the methods of treatment used and a discussion of their relative value in the control of glioma retinae.

TABLE VI.—*Methods of Treatment.*

| | Primary. | | Recurrence. (After enucleation.) | |
|---|---|---|---|---|
| | Unilateral. | Bilateral. | Orbital. | Intra-ocular. |
| Surgery alone | 3 | 1 | — | — |
| Surgery (one eye) + radiotherapy (post-operative or 2nd eye) | 3 | 7 | — | — |
| Radiotherapy alone | 1 | — | 1 | 3 |

A. *Primary cases.*

I. *Unilateral disease.*—Surgery alone was used in 3 cases, one of which showed optic nerve involvement. In 3 further cases surgery was followed by post-operative irradiation, in two of which there was evidence of extrabulbar spread. One case was primarily treated by radiation.

II. *Bilateral disease.*—The eye in which the disease was more advanced was excised in all 8 cases. The other eye was treated by radiotherapy—external radiation being used—in 7 of these. The remaining case was referred to us after the removal of one eye, for radiotherapy to the second eye. The disease even in this eye, however, had progressed so far that the sight could not have been saved; it was therefore decided to enucleate also this, the remaining eye.

B. *Recurrent cases.*

All the 4 recurrent cases were treated by radiotherapy. One case was seen with a recurrence in the socket 5 months after removal of the only affected eye. The other 3 cases had recurrences in the remaining eye following treatment respectively by insertion of radon-seeds into the eye, the insertion of radium needles into the orbit, or of diathermy puncture of the growth, the first eye having been excised.

### METHODS OF RADIOTHERAPY.

Prior to 1941 radium was used in our series as the source of irradiation; since then all cases have been treated by deep X-rays.

"*Primary*" *treatment.*

I. *Radium.*—Radium in the form of surface moulds was used in 2 cases for the treatment of the remaining eye. The dosage was calculated in mg.-hours, and has, for the purposes of this investigation, been recalculated—as far as was possible from the data available—in roentgens. It is interesting to note that the tumour dosage then applied was very similar in range to that used later in the X-ray-treated cases.

Two of the 3 bilateral cases who have survived 5 years were treated by this method. The actual survival times are 16 and 11 years. One case was treated on 3 occasions within 6 months, estimated tumour doses of 1440, 2900 and 3800 r. being applied until the growth was obviously shrinking. This boy can read large print. The case was previously reported by Mr. R. A. Greeves (1934). The other case was given an estimated tumour dose of 3000 r. (surface dose 5600 r.), and developed a posterior polar cataract, which is discussed below.

2. *Deep X-ray therapy.*—As long as radium moulds were being used it was inevitable that the whole of the globe was being irradiated to a high dose, but with the change-over to and use of small beams that were developed in deep X-ray therapy it became possible to limit the volume of high dose to a certain extent. Our technique has been directed at eradicating the focus of disease present rather than treating the whole of the posterior half of the retina (where the majority of the growths are situated). A recent case, however (not included in this survey), showed us that a certain risk is run thereby in that shortly after the treatment and subsequent disappearance of a small lesion below the equator by fields of 2 and 3 cm. diameter a further nodule appeared in the upper half of the retina.

In an endeavour to treat as small a part of the eye as possible we have used circular fields of 2 and 3 cm. diameter by means of special tubular applicators primarily intended for intrabuccal lesions as described by Roberts and Quick (1943). These are used in connection with our deep X-ray tubes, treatment being carried out at 190–220 kv. and focus-skin distances of 30 to 35 cm. A Thoraeus filter (1·0 mm. Al + 0·4 mm. Sn + 0·8 mm. Cu), giving a $\frac{1}{2}$-value layer of 2·1 mm. Cu, was used until 1942, since when, except in one case, filtration has been 0·5 or 0·8 mm. Cu, giving a $\frac{1}{2}$-value layer of 1·3 or 1·4 mm. Cu. Multiple fields are used, e.g. lateral (temporal), medial (transnasal), superior and inferior orbitals. The anterior field was abandoned as soon as it became apparent that the cornea was the most vulnerable structure of the eye, and disintegration of the eye had occurred in 2 or 3 cases in which this approach had been used. The lens, too, will receive a relatively large dose of irradiation when the anterior field is used and the lens dose should be kept at a minimum. We endeavour to ensure this by our technique of " by-passing " the lens as much as possible, which can be achieved in all the other fields mentioned.

*Positioning.*—Special problems of set-up arise because of the age of the patients and the small fields used so that accuracy of alignment is of much greater importance. Most of the children under the age of 4 need steadying during each treatment, and the older ones at least during the first 2 or 3 sessions. We have in a few cases tried to make individual plaster casts into which the child is put during treatment, the cast including a headpiece with a frontal process to obviate any possibility of head movement. In practice, however, even the smallest children have been found to wriggle around in these casts and human hands have had to help. The casts are useful, nevertheless, for steadying at least the trunk, and one escapes the otherwise necessary manoeuvre of binding the child on to the couch. In smaller children this can be done best by first wrapping the child tightly into a blanket, arms included.

All cases—and at each treatment—are set up by a medical officer, and during treatment, if necessary, the head steadied either by himself, by a radiographer, or by a nurse—or, if available, one of the parents. The usual precautions

(wearing of lead-apron and gloves and keeping out of the direct beam) are observed. This duty is taken in turn by a rota of assistants, so as to minimize exposure still further. In older children it may be possible after a few treatments to leave the immediate neighbourhood of the treatment couch and direct operations from a corner of the treatment cubicle, and later still from outside it. The installation of a two-way microphone-loudspeaker system helps with some children, but its use may scare others, and the whole approach must be a rather individual one.

*Dosage.*—Daily treatment are given, the daily dose varying from 100 r. to 300 r. to one or two fields, and our course has lasted from 3 to 6 weeks. The total tumour dose has varied, and depended partly on the progress observed ophthalmologically. Still more varied has been the total skin dose applied to individual fields. The highest was 4750 r. in 57 days. This maximum dose was 50 per cent higher than the 3100 r. and 3200 r. given to 2 fields of another case ; all other applied skin doses were below 2700 r., and most between 1000 and 2000 r. In many cases a moist desquamative skin reaction was produced, which healed within a month, leaving no lasting sequelae in the skin.

*Dosage and result.*—Successes have been obtained with deep X-rays by daily tumour doses of 80 r. as well as 200 r. and by treatments lasting 16 or 57 days, but the number of cases observed is too small to confirm the impression that neither dose rate nor the total time are of critical importance within a wide range. The total estimated tumour dose, however, showed a critical range : the successes were achieved by tumour doses of 3300 r. to 4400 r. irrespective of whether radium-moulds or deep X-rays were used, including one case in whom radium-moulds of increasing strengths were applied at short intervals within 5 months, the highest single dose being 3800 r.

Two main treatment schemes have been used : either a single " radical " course or repeated courses of deep X-rays, in each one of which a dose was given just sufficient to make the tumour disappear. This was usually achieved by doses of 2000–3000 r., to be repeated as soon as there was renewed activity. The latter scheme is somewhat similar to that recommended by Martin and Reese (1936, 1942, 1945), but in our hands it has never been successful, whereas the single radical course of treatment has given us outright cures.

We have only once had the opportunity of treating a unilateral case *ab initio.* This was a girl, aged 3 years and 9 months, who came with a history of over 2 years but still only a comparatively small growth. A total tumour dose of 2160 r. was given in 24 days and the lesion regressed. Ten months later, however, there was a large recurrence and the eye was excised. Histology confirmed the presence of active growth.

Sometimes it has been necessary to continue the first course up to a much higher tumour dose. There was one case in our series where even the second eye carried a growth covering almost the whole of the retina—he had come to us with simultaneous affection of both eyes. After the excision of the first eye, which was completely filled by growth, a tumour dose of 5730 r. was applied to the remaining eye. Within 60 days there was some regression, but definite activity was noticed again less than 4 months later and a further course of deep X-ray therapy started. This did not hold up the fatal issue and, in addition, there was recurrence on the enucleated side and massive secondary involvement of the liver (Case report 3).

In this part of the body as anywhere else—but perhaps even with greater force—is one struck by the confirmation of the hypothesis most prevalent for the explanation of failures through over-dosage. We know that in most of these cases the mechanism of healing is by calcification, a process presumably brought about by the connective-tissue stroma of the tumour bed—and occurring even spontaneously in a number of well authenticated cases (Hine, 1937). Over-dosage will damage the normal tissue—and the tumour bed—in such a way that it fails to produce the curative fibrosis and calcification ; or even when once started, this process may break down at some future date, and allow the growth to re-establish itself from a small number of still active cells or cell nests.

*Post-operative irradiation.*

In 3 cases, in 2 of which there was optic nerve involvement, post-operative irradiation was given after enucleation of the eye.

A radium mould was used in two cases and deep X-rays in the other. Two fields are used in the deep X-ray technique, an anterior and a lateral orbital, the field sizes being usually somewhat larger than in the primary technique, i.e. 5 cm., and the focus skin distance greater (40 cm.).

" Tumour " doses varied from 2560 r. (D.X.R.) to 3570 r. (Ra-mould), but in one case only 660 r. were apparently given (by Ra-mould), and this furnished the only recurrence in this group (Case 19). We have not as yet given post-operative treatment to the orbit of the removed eye in bilateral cases. This may be advisable as a general policy in view of the ultimate fate of some of them, i.e. local recurrence even where no apparent extra-ocular extension was originally present, unless one regards these deposits as due to retrograde extension from the second eye, which by that time has got completely out of control. One should also remember, as Duke-Elder (1940) points out, citing Meighan and Michaelson (1938), that there may be discontinuous spread in the nerve, so that the excised specimen (even if part of the nerve is included) need not necessarily show the extra-ocular extension which has in fact already taken place. In carrying out this suggestion the contribution that the lateral fields will make to the retained retina or the enucleated orbit respectively and which will amount to a depth dose of about 20 per cent must be taken into account. This technique has been used in a recent case which had a recurrence in the excised orbit as well as growth in the second eye, and is also applicable in the "prophylactic" irradiation of a case in which bilateral enucleation has been performed.

When we first collected these cases it appeared as if the unilateral cases treated post-operatively by irradiation would not be of very great interest because surgery alone might have been responsible for the good results. We have, however, in our series one case of recurrence within 6/12 of the operation (this was treated by a combination of deep X-rays and implantation of radon-seeds, but recurred again and proved rapidly fatal), and the above-mentioned case, which may be regarded as having been inadequately treated in the post-operative period. This case had a remarkably long latent period of 5 years, and a very large dose of irradiation was necessary to arrest the growth. The question of post-operative prophylactic irradiation in this disease should therefore still be regarded as an open one.

*Surgery.*

Surgical excision is the mainstay of therapeutic procedure in this condition. All bilateral cases in this series have had at least one eye excised—in one case which was sent to us for radiotherapy of the second eye the growth was already too advanced and this eye, too, was removed. (The child is alive without any sign of recurrence 5 years later.)

The three primary unilateral cases treated by surgery alone are still alive and well 3¾, 5¾ and 6 years later.

The type of operation performed consisted of an excision of the globe with as much of the optic nerve as possible.

*Recurrent cases.*

There were 3 cases in our series of recurrence after radium or radon implants or diathermy to growths in the remaining eye. All these were treated by deep X-rays. Two cases had several courses which failed to arrest the disease, and they have died 18 and 28 months after the first course. The third case, having been given 3400 r. tumour dose, developed chronic iritis and cataract within 4 months and the eye had to be removed. No growth was found on histological examination.

One case of massive recurrence in the orbit with bone involvement after enucleation of the only affected eye failed to respond to combined irradiation by deep X-rays and implantation of radon needles; another case, successfully treated, is of considerable interest. It is the case already mentioned which received a post-operative prophylactic dose of 660 r. by a radium mould and developed a recurrence 5 years later. The case illustrates several points of general radiological interest, and is of special importance as regards the natural history of this disease (Case report 19).

*Cataract.*

Radiation cataract, the commonest complication of radiotherapy of the eye, developed in 5 cases (45 per cent). One, having received 3400 r. by deep X-rays in 16 days for a recurrence after treatment by radon-seeds elsewhere (tumour dose estimated at 2000 r.), was complicated by chronic iritis and needed extraction of this the remaining eye 4 months later (now 5½ years ago)—no growth was found on histological examination, as already mentioned. The second case had 3300 r. also in 16 days, but did not develop cataract until 3 years later. This was needled recently. Another case developed its cataract 2½ years after receiving 4400 r. (in 57 days); this was extracted 4 years ago and vision is only partial now. Another cataract that was removed was obvious already 9 months after a dose of 3000 r. (given in 34 days at the age of 7 months), and the eye has since been excised for a recurrence of the neoplasm. The last case, treated by a radium mould (the dose in the lens lying between 5300 r. on the surface and 3000 r. estimated for the retina), developed a small posterior polar cataract not interfering with vision, 2½ years later. She received normal schooling, and is now—at the age of 15—able to read ordinary print, if with some effort.

The dose-time relationship together with the age of the child appear to determine the time of onset of the cataract. The 2 early cataracts (i.e. within less than 12 months of treatment) appeared in children irradiated at 7 and 16

months of age, whereas the comparatively " late " cataract was seen where treatment was given at the age of 2½ and 4 years. One other case in whom the daily tumour dose was less than 80 r. (at the age of 15 months) also developed a " late " cataract.

*Case reports.*

*Case* 3.—M. B—, a boy, 4 years of age, was first seen at the Middlesex Hospital on 29.i.41 with a history that 7 months previously his mother had noticed a white spot in the right eye and 5 months later similarly in the left eye. His general condition was good, but he was rather drowsy. Rt. eye: whole eye filled with whitish neoplastic mass extending down from medial part of retina. Small portion of normal retina seen. Lt. eye : Perception of light and ?? counts fingers.

3.ii.41 : Excision of right eye (Mr. M. H. Whiting). Histology : Glioma retinae of retinoblastic type. Optic nerve involved.

4.ii–2.iv.41 : Course of deep X-rays (25 treatments) to left eye, using 4 fields (lat. 2700 r., ant. 1400 r., sup. 1800 r., inf. 1500 r.), each 3 cm., at 200 kv., 10 Ma., Thoraeus filter, ½-val. layer 2 mm. Cu, F.S.D. 35 cm. Tumour dose 5727 r.

Meanwhile, on 17.ii.41 the child began to be brighter, and on 3.iii.41 the growth appeared smaller. 13.v.41 : Lt. eye looks improved. Counts fingers. 8.vii.41 : Sight better. O.E. : Shallow detachment temporal side and some shrinking of growth on nasal side. 1.viii.41 : Seeing less well, but distinguishes light and dark. O.E. : Most of retina shows dead white.

2–15.viii.41 : Further course of D.X.R. to 3 fields (same technical factors). Tumour dose 2785 r.

9.xii.41 : Readmitted to Sector Hospital. Very pale, unhappy, miserable. Vision : Can see light and crude shapes. No light reflex. Three weeks later he developed an enlarged liver and severe anaemia (Hb 25 per cent., R.B.C. 1·7 m.) due to rupture of the liver and resultant haemoperitoneum. Haemoperitoneum has not to my knowledge previously been reported in the literature as a complication of hepatic metastases due to retinoblastoma.

4.i.42 : Died.

P.M. findings: *Skull.*—Many extradural haematomata (secondaries ?). *R. orbit.*—(Eye excised 11/12 previously). Bone eroded into sella turcica. Right greater wing of sphenoid dark red and friable; the red friable mass extends as far as the left orbit but not into it. *L. eye.*—(Treated by D.X.R. 5 and 11 months previously.) Some of the muscles of the eye red and haemorrhagic. *Abdomen.*—As above. *Histology.*—Diffuse infiltration of the left eye, bones, and liver with retinoblastoma (i.e. similar to the original histology).

*Case* 14.—J. L. H— first came under observation at the Middlesex Hospital in July, 1945, at the age of 9/12, having had his left eye excised elsewhere 3 months before (histology showed retinoblastoma without involvement of the optic nerve), and at the same time a focus the size of a disc in the periphery of the right retina had been isolated by diathermy barrage. At this time there was no recurrence in the socket, but two new foci of growth had appeared in the right eye. He was given a course of deep X-rays (T.D. 1750 r.), and when in February, 1946, there was again obvious activity a further 3000 r. was given.

1.

2.

FIG. 1 and 2.—Case 19, D. M—, 17.x.46. Showing calcified (healed) glioma retinae in right orbit.

Japha.

Eight months later the old foci looked less active and more solid. About a fortnight after this examination the boy began to vomit (apparently in a rather projectile manner) and became drowsy. When examined again on 20.xi.46 there were signs of brain-stem involvement, and a course of deep X-rays directed at this region was started. There was some temporary improvement, but the child died within 3 months of the onset of the final stage. Lumbar puncture during this time showed the presence of peculiar mononuclear cells in the cerebrospinal fluid, which in the light of the later post-mortem findings must be regarded as retinoblasts. A section of the midbrain revealed a column of growth in the aqueduct. Besides this there was, on histological examination, active growth in the right eye with involvement of the optic nerve on this side as well as in the stump of the left optic nerve, and in the pia arachnoid over the cerebral hemispheres and midbrain. Multiple small secondary nodes were present in the liver.

*Case* 19.—D. M—, a boy, 5 years of age, was sent to the Middlesex Hospital in 1929 from Jersey, where 5 months previously his right eye had been removed for glioma retinae. A radium mould was applied as a "delayed post-operative measure," but the surface dose when recalculated recently was found to have been only 660 r. (in 36 hours). Nearly 5 years later there was a large swelling in the orbit which was treated by X-rays in Jersey (details unknown) and the swelling disappeared. A few months later a further course of X-rays was given for a "small hard bean-shaped swelling" in the lower half of the orbit.

Seven months after this the mass was again thought to be larger and the boy referred back to the Middlesex Hospital in September, 1936. He was found to have "a hard mass 2 cm. in diameter protruding from the inferior wall into the orbit." Treatment was given by a nidrose and elastoplast radium applicator (radium skin distance 3 cm., area 56 cm.$^2$, charge $2 \times 6{\cdot}6 + 3 \times 10 + 2 \times 13{\cdot}3 = 69{\cdot}8$ mg., filtration 1 mm. Pt., time 283 hours) and in 2 weeks the eyelids received a dose of 11,000 r., the estimated dose at the posterior orbital wall being 6000 r. The boy was lost sight of during the war, but reported again as a young man in October, 1946, that is, 10 years later, when he presented with marked telangiectases on the right cheek around the orbit and a small sinus over the zygomatic arch. A skiagram showed a certain amount of bone necrosis in the frontal and malar bones and also an irregular calcified mass in the orbit (Fig. 1 and 2). The other eye has remained normal.

SUMMARY.

The literature on the treatment of glioma retinae is reviewed, and 19 cases seen at the Middlesex Hospital between 1929 and 1946 are described as regards age and sex incidence, length of history, side affected, family history, pathology, including some remarkable post-mortem findings, and their treatment discussed in detail. The results of radiotherapy are summarized in Table VII.

(a) *Post-operative irradiation* has been successful in the two cases where adequate treatment was given irrespective of whether a radium mould or deep X-rays were applied. In the one case described at length above, the first treatment, where 660 r. only were given, must be regarded as quite inadequate, but the recurrence which developed 5 years later responded well to a radical course.

(b) *Primary irradiation.*—The two cases treated by radium moulds and two of the three cases treated by deep X-rays more than 5 years ago are alive and well 15, 10, 5 and 5 years after treatment. Cataract has developed in three of these cases and two have had operations for its removal. The sight in these three cases is partial only; the fourth case has normal sight. A minimum tumour dose of 3300 r. is considered essential, and overdosage (which may lie at anything more than 4500 r.) is warned against.

I wish to express my grateful thanks to Mr. Greeves, Mr. Whiting and Mr. Goldsmith, under whose care many of these cases were admitted, and whose ophthalmological examinations have formed the basis upon which the radiotherapist has been able to build. My special gratitude belongs to Professor Windeyer, who directed the treatment of all the radiotherapeutic cases, for his unfailing encouragement and many valuable suggestions.

TABLE VII.—*Results of Radiotherapy* (15 *Cases*).

| | Primary Cases (11). Treated. | | | Recurrent Cases (4). Treated. | | | Total. | |
|---|---|---|---|---|---|---|---|---|
| | More than 5 years ago. | | Less than 4 years ago. | More than 5 years ago. | | Less than 4 years ago. | | |
| | Alive and well. | Died. | Alive and well. | Alive and well. | Died. | Died. | Alive and well. | Died. |
| Bilateral | 4 | 1 | 2(*a*) | 1(*b*) | 1 | 1 | 7 | 3 |
| Unilateral | 3* | — | 1(*a*) | — | 1 | — | 4 | 1 |
| Total | 7 | 1 | 3 | 1 | 2 | 1 | 11 | 4 |

(*a*) (*b*) Eye excised following failure of radiotherapy for (*a*) recurrence, (*b*) chronic iritis and cataract.

* Post-operative irradiation. One case lost sight of after 33 months. One case treated for recurrence 5 years later—now 12 years ago.

### REFERENCES.

CUMMINGS, J. N., AND SORSBY, A.—(1944) *Brit. J. Ophthal.*, **28**, 533.

DUKE-ELDER, W. S.—(1940) 'Textbook of Ophthalmology,' vol. iii, p. 2826. London (Kimpton).

GREEVES, R. A.—(1934) *Trans. ophthal. Soc. U.K.*, **54**, 420.

GRIFFITH, A. D., AND SORSBY, A.—(1944) *Brit. J. Ophthal.*, **28**, 279.

HINE, M.—(1937) *Trans. ophthal. Soc. U.K.*, **57**, 173.

MARTIN, E. H., AND REESE, A. B.—(1936) *Arch. Ophthal N.Y.*, **16**, 733.—(1942) *Ibid.*, **27**, 40.—(1945) *Ibid.*, **33**, 429.

MEIGHAN, S., AND MICHAELSON, I. C.—(1938) *Trans. ophthal. Soc. U.K.*, **58**, 208.

MOORE, R. F.—(1930) *Proc. Roy. Soc. Med.*, **23**, 475.—(1935) *Trans. ophthal. Soc. U.K.*, **55**, 1.

PARKHILL, E. M., AND BENEDICT, W. L.—(1941) *Amer. J. Ophthal.*, **24**, 1354.

PHILPS, S., AND HOULTON, A. C. L.—(1944) *St. Bart.'s Hosp. J.*, **47**, 278.

RAY, B. S., AND MCLEAN, J. M.—(1943) *Arch. Ophthal. N.Y.*, **30**, 437.

REESE, A. B.—(1931) *Ibid.*, **5**, 269.—(1940) In 'Treatment of Cancer and Allied Diseases,' ed. Pack, G. T., and Livingston, E. M., vol. iii, p. 2150. New York (Hoeber).

ROBERTS, J. E., AND QUICK, R. S.—(1943) *Brit. J. Radiol.*, **16**, 82.

STALLARD, H. B.—(1933) *Brit. J. Ophthal.*, Monograph Suppl. VI.—(1948) *Ibid.*, **32**, 618.